# Racial Disparities in Incidence of Diaper Dermatitis and Implications for Diaper Inequities

**DOI:** 10.1089/heq.2024.0083

**Published:** 2024-09-26

**Authors:** Jennifer Randles, Justin van Zerber, Kristian Browning, Balaraman Rajan, Benito Delgado-Olson

**Affiliations:** ^1^ College of Social Sciences, California State University, Fresno, California, USA.; ^2^ SupplyBank.org, Oakland, California, USA.; ^3^ Department of Management, California State University, Hayward, California, USA.

**Keywords:** diaper dermatitis, health disparities, pediatric health, public health, racial minority

## Abstract

**Objectives:**

To examine if the likelihood of infants and toddlers requiring medical care for diaper dermatitis, a condition linked to infrequent diaper changes and possible diaper need, is different across racial/ethnic groups.

**Materials/Methods:**

This is a population-based retrospective study. We collected data via public records requests from the California Department of Health Care Access and Information to determine the number of patients aged 0 to 3 years diagnosed with diaper dermatitis from 2010 to 2021 in emergency departments (EDs) and hospitals. We used two sample t-tests to compare the incidences of ED visits and in-patient hospitalization during the 11-year time period to identify differences across racial/ethnic groups.

**Results:**

From 2010 to 2021, there were 184,097 total diagnoses of diaper dermatitis, 53,678 of which received in-patient treatment. The annual mean was 15,341 and ranged between 9,407 and 17,425. The prevalence (per 1,000 children aged 0–5 of the respective race) was highest for the Black population averaging 9.56 (range: 5.79 to 11.37). The prevalence was 5.93 (3.75 to 7) for the White population, 2.49 (1.78 to 3.14) for Asian/Pacific Islanders, and 6.67 (4.25 to 7.52) for the Hispanic population. We find that Black children were disproportionately diagnosed with diaper dermatitis in California hospitals and EDs (*p* < 0.001).

**Conclusions:**

Racial disparities in medical conditions that can result from lack of sufficient diapers point to diaper need as a social determinant of health deserving of greater public attention and policy redress.

## Introduction

Infrequent diaper changes, diapers made from nonbreathable materials, and ill-fitting diapers all increase the risk of childhood dermatological conditions, including diaper dermatitis or what is more commonly known as “diaper rash.”^1–2^ Diaper dermatitis, inflammation localized to the skin area covered by a diaper, can have many causes, including infections, friction irritation, chemical allergies, sweat, decomposed urine, and plugged sweat glands. Most cases of infantile diaper rash are irritant contact dermatitis caused by prolonged contact with urine and feces.^3^

Diaper dermatitis in young children is a primary reason for many pediatric office and emergency department (ED) visits each year.^4–5^ Primary advice for how to prevent diaper dermatitis in the first place is to change wet and soiled diapers as quickly and frequently as possible.^6–7^ Frequent changing of superabsorbent diapers that wick wetness away from a child’s skin is found to be associated with a lower incidence of diaper dermatitis.^8^

A study by Ward et al. totaling outpatient visits for diaper dermatitis across the United States from 1990 to 1997 found no disproportionate diagnoses by infant sex, race, or ethnicity.^4^ It also found that most cases of diaper dermatitis go untreated by a physician, as fewer than 10% of episodes are referred for treatment, suggesting that official counts of diaper dermatitis vastly underestimate its actual prevalence. The prior research on demographic predictors of diaper dermatitis has focused on age, sex, and race/ethnicity, finding that female and male infants and infants of all races are proportionately diagnosed during outpatient visits.^9–10^

Some evidence suggests that diaper dermatitis is linked to inadequate access to diapers that allow for frequent diaper changes. Sobowale, Clayton, and Smith found that parents who reported utilizing organizations that distributed diapers or experiencing diaper need—the inability to afford and access enough diapers to keep a child dry, comfortable, and healthy—were more likely to have sought pediatric care for diaper dermatitis.^11^

However, existing research focused on pediatric office visits for conditions linked to infrequent diaper changes and possible diaper need did not explore differences in the likelihood of seeking ED care, another key source of pediatric care for diaper dermatitis. This study therefore aims to examine if the population prevalence of requiring emergency medical care for diaper dermatitis is different based on race/ethnicity, a likely predictor of diaper need given high rates of racialized childhood poverty.^12^

## Materials and Methods

We obtained data via public records requests from the California Department of Health Care Access and Information (HCAI) to identify all patients aged 0 to 3 years diagnosed with diaper dermatitis from 2010 to 2021 during ED visits and in-patient hospitalizations. HCAI integrates and centralizes data from all hospitals licensed by the California Department of Public Health. All 308 California EDs in non-suspense status that saw patients are represented in the data. For privacy reasons, the data does not contain patient-level identifiers and hence repeated visits cannot be traced to any given patient, nor does it differentiate between primary and secondary diagnoses. ICD-9-CM (International Classification of Diseases, Ninth Revision, Clinical Modification) and ICD-10-CM (International Classification of Diseases, Tenth Revision, Clinical Modification) diagnosis codes for diaper dermatitis—(ICD-9-CM) 691.0 and (ICD-10-CM) L22—were used to identify patients categorized by condition and source of care (ED or in-patient treatment). The dataset used as the basis of this analysis was deidentified before receipt and therefore does not constitute human subjects research nor require IRB approval.

These demographics were subcategorized by age, sex, race, preferred language, and expected payer. From 2010 to 2021, HCAI collected racial and ethnic data using the following options: American Indian/Alaska Native, Asian/Pacific Islander, Black, Hispanic, White, and All Other (including multirace/unknown/invalid). HCAI did not include other common racial/ethnic identifiers, such as Latinx and Indigenous Person.

We used two sample t-tests to compare the incidences of ED visits and in-patient hospitalization from 2010 to 2021 for diaper dermatitis across racial/ethnic groups to identify disproportionality. The total number of children aged 0 to 5 by year in California from the US Census was used to calculate the prevalence per 1,000 children of the respective race.

## Results

From 2010 to 2021, there were 184,097 presentations of diaper dermatitis among children under 3 years presenting to emergency departments and of these 53,678 received in-patient treatment. The annual mean was 15,341 cases with a steady decline in total number of cases beginning in 2017. During 2020 and 2021, the total annual number of reported cases dropped by 38% when compared with the annual average of the previous decade. See Table 1 for year-wise diagnoses of diaper dermatitis and Table 2 for diagnoses documented by race/ethnicity.

**Table 1.** Diagnoses for Diaper Dermatitis for Children Aged 0 to 3 Years Presenting to the Emergency Department 2010–2021

Year	Emergency room visits	Inpatient visits	Total
2010	11,331	4,178	15,509
2011	12,474	4,193	16,667
2012	13,274	3,984	17,258
2013	12,784	4,566	17,350
2014	13,159	4,266	17,425
2015	12,096	4,318	16,414
2016	12,530	4,651	17,181
2017	11,143	4,848	15,991
2018	10,282	4,983	15,265
2019	9,895	4,904	14,799
2020	5,094	4,313	9,407
2021	6,357	4,474	10,831
Total	130,419	53,678	184,097

**Table 2.** Total California Diagnoses of Diaper Dermatitis 0–3 Years by Race/Ethnicity 2010–2021

Year	All other race, inc multi/unk/inv	American Indian/Alaska native	Asian/pacific islander	Black	Hispanic	White	Total
2010	948	38	529	1,278	8,674	4,042	15,509
2011	933	30	584	1,438	9,326	4,356	16,667
2012	926	33	597	1,494	9,700	4,508	17,258
2013	944	53	698	1,499	9,827	4,329	17,350
2014	994	63	686	1,531	9,884	4,267	17,425
2015	1,044	58	727	1,321	9,265	3,999	16,414
2016	1,136	78	826	1,443	9,608	4,090	17,181
2017	1,191	57	822	1,286	8,762	3,873	15,991
2018	1,179	37	884	1,237	8,398	3,530	15,265
2019	1,353	51	847	1,088	8,197	3,263	14,799
2020	892	33	561	698	5,038	2,185	9,407
2021	1,019	26	595	775	5,968	2,448	10,831
Total	12,559	557	8,356	15,088	102,647	44,890	184,097

There were statistically significant differences (*p* < 0.001) in the frequency of diagnosis among hospitalizations and ED visits across different racial and ethnic groups (see Table 3). In California, the prevalence of diaper dermatitis was higher among Black patients (9.56 per 1,000 children) than among White patients (5.93 per 1,000 children) and Asian/Pacific Islanders (2.49 per 1,000 children). Hispanic (6.67 per 1,000 children) patients had a slightly higher prevalence than the White population.

**Table 3.** *p*-Values from *t*-Tests for Race and Ethnicity Comparison (12 Observations 2010–2021)


	Black patients	Hispanic patients	White patients	Asian/Pacific Islander
Average prevalence (per 1,000 children)	9.56	6.67	5.93	2.49
Hispanic patients	<0.001		0.084	<0.001
White patients	<0.001	0.084		<0.001
Asian/Pacific Islander	<0.001	<0.001	<0.001	

We performed the same tests by removing 2020 and 2021 due to the significant decrease in the number of visits—likely due to COVID-19 related constraints and restrictions—and received very similar results. In addition, we performed these tests including data for two related pediatric urological and dermatological conditions—urinary tract infections and candidiasis infections—and obtained similar results. Those results are available upon request.

## Conclusions

Study results revealed that Black children were significantly more likely to present to EDs with diaper dermatitis, a dermatological condition potentially associated with prolonged diaper wear, than other racial groups. Hispanic children had a slightly higher prevalence of diaper dermatitis diagnoses than White children. Given high rates of racialized childhood poverty among Black and Hispanic children,^12^ this suggests that racial/ethnic factors, especially racialized differences in income and access to health resources, are related to the proportion of infants and toddlers who receive urgent or hospital medical care for skin-related health problems associated with inadequate access to diapers and other health resources.

## Discussion of Healthy Inequity Implications

Diaper need, the inability to afford adequate diapers, affects one in three families in the United States where high childhood poverty rates are associated with lacking basic needs.^13–14^ Almost half of U.S. infants and toddlers are in families that live at or below 200% of the federal poverty level, and 10% of children 3 and younger live in deep poverty at less than 50% of the poverty threshold.^15^ Children of color are significantly more likely to be among these groups, as 34% of Black children and 28% of Hispanic children live in poverty, compared with only 11% of White and Asian children.^12^

Diaper need is an adverse childhood experience linked to racialized poverty and worse mental and physical health for both children and parents.^16–20^ Mothers of color report racialized diaper-related stigma, stress, and surveillance, suggesting that diaper need contributes to racial disparities in maternal and infant health.^21,22^ Previous research reveals that parents cope with diaper need by using “diaper stretching” strategies associated with diaper dermatitis.^18,19^ In one study, 8% to 28% of parents surveyed reported prolonged diaper wear as a diaper need management strategy; more than 20% reported using other strategies associated with diaper dermatitis, including using other nonbreathable household items for diapers, such as towels or cloth, and using diapers that are too big or too small.^18^ Previous qualitative research found that more than half of mothers experiencing diaper need use these strategies and that stigma and fear of child welfare system involvement may lead to underreporting of diaper-stretching strategies to researchers.^19,21^

Given the connection between the risk of pediatric dermatological conditions and common diaper-stretching strategies, it is important to examine potential disparities in diagnosis of health conditions possibly linked to diaper need while receiving hospital-based medical care. To prevent and treat dermatological conditions associated with diapering, experts recommend parent support and education about general skin care including more frequent diaper changes, choosing more breathable diapers, and using topical barrier creams.^6,23,24^ These recommendations assume that parents have access to sufficient resources to change diapers more frequently, choose among different diapering options, and afford additional diapering supplies. As prior research suggests, the problem is not lack of knowledge or care among economically vulnerable parents, but rather inequitable access to diapers.^18,19^

Diaper distribution organizations are one source of support. However, despite increasing service capacity, diaper banks rarely receive enough funding to meet all community needs. They must rely primarily on private and in-kind donations, and their services do not cover all geographic areas. Diaper/supply/basic needs banks distributed 52 million diapers to more than 277,000 children in 2016 yet met only 4% of the estimated diaper need.^25^ Public programs, including the Special Supplemental Nutrition Program for Women, Infants, and Children, do not cover diapers, and cash aid benefits are often too low to cover the cost of diapers along with other basic needs. The $100 average monthly diaper bill for one infant would use 9–49% of the average monthly state cash aid benefit for a family of three through Temporary Assistance for Needy Families.^26^

Our study results point to a link between race and seeking hospital-based medical care for a pediatric health problem associated with diaper need. The greater likelihood that children of color will be diagnosed with diaper dermatitis suggests that diaper need is a social determinant of health deserving of greater public attention and policy redress. Though public support for community diaper distribution is growing, this added investment is still insufficient to meet community needs, and piecemeal public funding reliant on legislative fiscal renewal can compromise community trust when grant-dependent organizations cannot guarantee sustained assistance to parents who depend on diaper support. Therefore, sustainable public solutions are needed to guarantee consistent, universal diaper support for families in need, especially as an issue of racial and economic equity.

Although results reveal significant relationships between race and the likelihood of being diagnosed with diaper dermatitis in EDs and hospitals, available data did not allow us to determine causation. We were unable to ascertain how many cases were directly attributable to insufficient diapers or if the families of children diagnosed with diaper dermatitis were experiencing diaper need. Moreover, although the practices of stretching diapers or reusing diapers may result in diaper dermatitis, not all families who experience diaper need use these methods. However, prior research finds that a significant portion do.^18,19,21^

Also, these numbers represent the total frequency of diaper dermatitis diagnoses, not total patients treated; patients may have been counted more than once if they presented with diaper dermatitis more than once during the study period. It is also possible that patients treated through EDs and in-patient care for diaper dermatitis were initially admitted for other conditions, as the data do not differentiate between primary and second diagnoses. Moreover, observed racial differences may not be attributable to differential diaper access but rather racialized differential care-seeking patterns (such as increased ED visits among Black children for other conditions), access to outpatient primary care, or a combination of these factors. Still, infants diagnosed with diaper dermatitis alone or associated with other health issues were more likely to be children of color. Whether preventative or curative, part of the remedy is adequate access to breathable, well-fitted diapers that enable frequent diaper changes.

These study limitations and findings suggest that future research should directly identify the prevalence and degree of diaper need among families that present in EDs and for in-patient treatment for pediatric diaper dermatitis. This could entail asking about diaper access on intake forms and offering information about diaper support services during the discharge process. Given the high costs of publicly funded ED visits and hospitalizations to taxpayers, this research also points to public investment in diaper support for socioeconomically vulnerable families as a fiscally responsible cost-saving measure and prudent strategy for mitigating one of the toxic effects of racial and economic inequalities on pediatric health.

## Abbreviations Used

CD-9-CM: International Classification of Diseases, Ninth Revision, Clinical Modification
ED: emergency departments
HCAI: California Department of Health Care Access and Information
ICD-10-CM: International Classification of Diseases, Tenth Revision, Clinical Modification
